# Genetic Variants Associated with Long-Terminal Repeats Can Diagnostically Classify *Cannabis* Varieties

**DOI:** 10.3390/ijms232314531

**Published:** 2022-11-22

**Authors:** Jackson M. J. Oultram, Joseph L. Pegler, Greg M. Symons, Timothy A. Bowser, Andrew L. Eamens, Christopher P. L. Grof, Darren J. Korbie

**Affiliations:** 1 Centre for Plant Science, School of Environmental and Life Sciences, College of Engineering, Science and Environment, University of Newcastle, Callaghan, NSW 2308, Australia; 2 Extractas Bioscience, 160 Birralee Road, Westbury, TAS 7303, Australia; 3 Impact Science Consulting, 24 Leighton Bay Drive, Metung, VIC 3904, Australia; 4 School of Health and Behavioural Sciences, University of the Sunshine Coast, Sippy Downs, QLD 4556, Australia; 5 Centre for Personalised Nanomedicine, Australian Institute of Bioengineering and Nanotechnology, The University of Queensland, St. Lucia, QLD 4072, Australia

**Keywords:** *Cannabis sativa* (*Cannabis*), genetic diversity, genetic resolution, population structure, reduced representation shotgun sequencing, single nucleotide polymorphism (SNP), long terminal repeat (LTR) retroelements

## Abstract

*Cannabis sativa* (*Cannabis*) has recently been legalized in multiple countries globally for either its recreational or medicinal use. This, in turn, has led to a marked increase in the number of *Cannabis* varieties available for use in either market. However, little information currently exists on the genetic distinction between adopted varieties. Such fundamental knowledge is of considerable value and underpins the accelerated development of both a nascent pharmaceutical industry and the commercial recreational market. Therefore, in this study, we sought to assess genetic diversity across 10 *Cannabis* varieties by undertaking a reduced representation shotgun sequencing approach on 83 individual plants to identify variations which could be used to resolve the genetic structure of the assessed population. Such an approach also allowed for the identification of the genetic features putatively associated with the production of secondary metabolites in *Cannabis*. Initial analysis identified 3608 variants across the assessed population with phylogenetic analysis of this data subsequently enabling the confident grouping of each variety into distinct subpopulations. Within our dataset, the most diagnostically informative single nucleotide polymorphisms (SNPs) were determined to be associated with the long-terminal repeat (LTRs) class of retroelements, with 172 such SNPs used to fully resolve the genetic structure of the assessed population. These 172 SNPs could be used to design a targeted resequencing panel, which we propose could be used to rapidly screen different *Cannabis* plants to determine genetic relationships, as well as to provide a more robust, scientific classification of *Cannabis* varieties as the field moves into the pharmaceutical sphere.

## 1. Introduction

*Cannabis sativa* (*Cannabis*) is one of the earliest cultivated plants with evidence of domestication dating back to the early Neolithic period, around 12,000 years ago. Recent evidence places these early cultivation events in the south east regions of China, prior to movement of *Cannabis* derived products across the globe in association with nomadic groups, leading to the widespread cultivation of *Cannabis* observed today [1,2]. *Cannabis* exhibits substantial versatility, with various parts of the plant providing fiber, and food, as well as intoxicant and medicinal molecules. These medicinal compounds are secondary metabolites, specifically the cannabinoids, produced in glandular trichomes that protrude from the epidermal layer, predominantly on the floral organs of female plants [3]. The most well-known and comprehensively studied cannabinoids are Δ-9-tetrahydrocannabinol (THC) and cannabidiol (CBD). THC is the molecule responsible for the psychoactivity or ‘high’ reported by *Cannabis* users when it modulates endogenous cannabinoid receptors in the central nervous system and peripheral tissues. In mammals, CBD also binds to endogenous receptors, however unlike THC, CBD receptor binding does not elicit a psychoactive response. Instead, CBD has been reported to mediate medically beneficial outcomes, such as its use in the prevention of epileptic seizures [4,5]. In addition to THC, CBD, cannabigerol (CBG) and cannabichromene (CBC), more than 100 other ‘minor’ cannabinoids are produced by the *Cannabis* plant [6]. However, in contrast to the major cannabinoids, THC, CBD, CBG and CBC, the medicinal properties of the minor cannabinoids produced by the *Cannabis* plant remain largely unknown, and therefore, when considered together, potentially represents a grossly underutilized natural medicinal resource.

Thousands of available *Cannabis* varieties have been developed from historical illicit cultivation and which now form the basis of the developing medicinal *Cannabis* industry. Typically, ‘new’ varieties are claimed following the cross breeding of two existing varieties, considered as genetically distinct to one another, with subjectively superior progeny selected for further cultivation. Selection may be based on phenotypic or chemotypic characteristics, with high THC or CBD often preferentially selected as desirable traits. The delineation between what is termed ‘hemp’ and ‘marijuana’ is based on the overall percentage (%), weight by weight (*w*/*w*) content of THC in dried flowers; below 1.0% for hemp classification in some Australian jurisdictions, though the number varies internationally. Suggestions have been made to classify *Cannabis* based upon geographic origin, phenotype or secondary metabolite profile [7], yet domestication, gene flow, and strong selection for singular attributes obscure both the scientific consensus and the utility of these approaches pursued in isolation. Additionally, recreational *Cannabis* culture frequently uses the terms ‘*sativa*’ and ‘*indica*’ to describe different varieties based on a suggestion of distinct subspecies, or in a sense to describe user ‘high’. Plant breeding programs based solely on these individual qualities, such as metabolite profiles, may in time drive desirable traits to stabilization, however, little to no scientific standard or literature exists outlining the qualifiers for novelty of a *Cannabis* variety, and by extension, when new variety nomenclature is appropriate.

A greater stringency applied to varietal classification would greatly strengthen the legitimacy of the industry, particularly in a clinical context in which dried flower, THC and/or CBD is prescribed. Metabolite analysis can be used as an effective strategy for *Cannabis* variety identification as well as to provide information on cannabinoid content through several analytical platforms, including high performance liquid chromatography (HPLC) [8], UV coupled reverse-phase HPLC [9,10], or spectroscopy-based detection [11,12]. However, discrepancies can still arise through variation in extraction protocol and analytical method used. Moreover, cannabinoid content from plant to plant of the same variety, or of progeny plants cultivated by different growers may vary considerably. For example, both the overall yield and cannabinoid content of *Cannabis* will be influenced by the growth environment, with yield and cannabinoid profile reported to vary in response to light intensity, light quality, nutrient availability, and plant density [13]. Given the variability inherent in intra-variety production of THC/CBD and the extent to which different growth conditions can alter cannabinoid profiles, a more robust method of classification is required. While metabolite analysis is integral to industry regulation and variety classification, supporting genomics could enhance regulation by identifying distinct varieties based on polymorphisms at the DNA level, thereby reducing susceptibility to change from environmental variation than what is observed when using a metabolite profiling approach.

**Figure 1:**
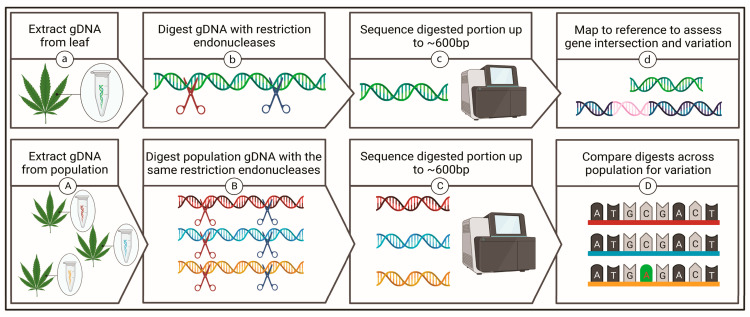
Simplified overview of two complexity reduction approaches. (**a**) Genomic DNA is extracted and (**b**) digested with restriction endonucleases prior to (**c**) sequencing and (**d**) mapping to a publicly available reference assembly. The use of a reference sequence allowed assessment of fragments able to be mapped to known coding sequences or genetic features. A slightly varied application requires the (**A**) extraction of genomic DNA from a population which is then (**B**) digested prior to (**C**) sequencing, after which sequences are (**D**) compared to one another to assess geneticvariation of isolated fragments across the population without the use of a reference assembly. Created with BioRender.com accessed on 17 August 2022.

Within this study, an analysis of 10 *Cannabis* varieties was performed using a reduced representation shotgun sequencing approach to identify genetic variants associated with CBD and THC levels (Figure 1). Principal component analysis (PCA) and discriminant analysis of principal components (DAPC) was subsequently employed to determine variety clustering, from which a minimal set of highly diagnostics SNPs were identified as able to effectively distinguish between the different varieties. As medicinal *Cannabis* moves rapidly into a highly regulated clinical setting, these SNPs have the potential to be incorporated into reduced cost sequencing methods alongside standardized metabolomics, to provide for precise determination of distinct *Cannabis* varieties in an evolving sphere which continues to lack detailed genomic information.

## 2. Results and Discussion

### 2.1. In-Silico Modeling and Selection of Restriction Endonuclease Combinations for Complexity Reduction

**Figure 2:**
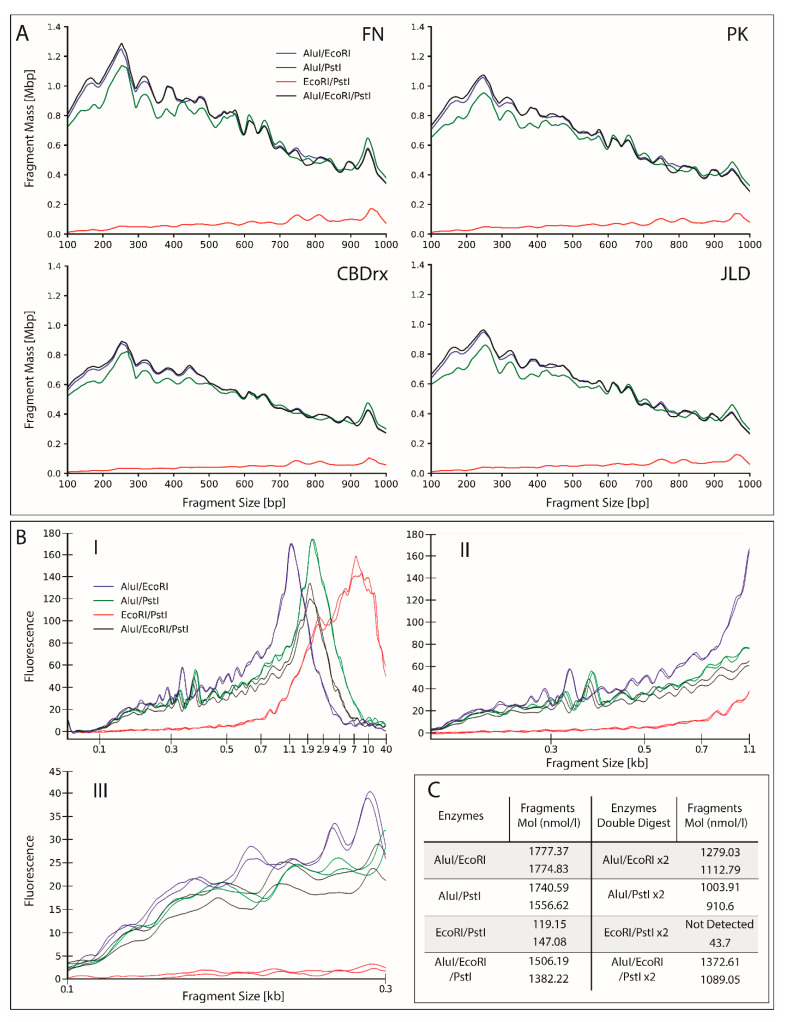
Digestion of *Cannabis* assemblies and Swiss Dream Auto genomic DNA. (**A**) Four *Cannabis* assemblies, Finola (FN), Purple Kush (PK), CBDrx (CBD), and Jamaican Lion DASH (JLD), were digested in silico with four restriction endonuclease combinations, including the AluI/EcoRI, AluI/PstI, EcoRI/PstI, and AluI/EcoRI/PstI combinations. (**B**) *Cannabis* genomic DNA was digested with the same four restriction endonuclease combinations and the fragmentation profiles were assessed; (**I**) up to 40 kb; (**II**) between 100 and 1000 bp and; (**III**) between 100 and 300 bp on a LabChip Bioanalyzer to determine the optimum combination. (**C**) Smear analysis was performed to compare single (left panel) and double (right panel) digestion reactions in replicate to determine the degree of ‘completeness’ of each restriction endonuclease combination.

To assess fragmentation profiles prior to lab-based complexity reduction and to determine the best combination of restriction enzymes to use, publicly available *Cannabis* genome assemblies were digested in-silico and subsequent fragment profiles modelled. All possible combinations of the selected restriction endonucleases, *Alu*I, *Eco*RI and *Pst*I, were assessed. The resulting in-silico profiles were then compared to *Cannabis* Swiss Dream Auto genomic DNA (gDNA) samples which were physically treated using the same enzymes. The resulting fragment profiles were then analyzed using Labchip GX’s digital electropherogram. Figure 2 presents the data obtained from both approaches, with a specific focus on digestion fragments between 100 to 1000 base pairs (bp) which proved to more accurately reflect the fragment pool applicable to short-read sequencing. Of note, the LabChip electropherogram uses a Savitzky-Golay filter for data smoothing, also known as locally weighted scatterplot smoothing (LOWESS), which was integrated into the developed Python script to reduce noise, and therefore, smooth the data. The modeling presented here maintains X and Y axis linearity, while the LabChip electropherogram exhibits a non-linear X axis that compresses the larger sized fragments which caused the large peak of nucleic acid bulk observed for fragments above 1000 bp in length, and which also reflects the gel electrophoretic mobility of nucleic acid fragments. For whole genome digest context, 1 to 10 kilobase (kb) fragmentation profiles were modeled separately (Figure S1). 

Across the assemblies, similar profile trends were evident when comparing the different restriction endonuclease combinations. More specifically, the *Alu*I/*Eco*RI and *Alu*I/*Eco*RI/*Pst*I combinations show similarities to one another across each assembly, with only slight separation between profile lines from 100 to around 400 bp, which reduces to the point of overlap through to 1000 bp (Figure 2A). The relatedness of these two profiles suggests that *Pst*I does not contribute significantly to restriction fragment production in this fragment size range. These two combinations also have the largest fragment mass up to approximately 500 bp, beyond which the *Alu*I/*Pst*I combination largely matches their profile. While the peak profiles of *Alu*I/*Pst*I follow that of the *Alu*I/*Eco*RI and *Alu*I/*Eco*RI/*Pst*I profiles, the modeling suggests a reduced fragment mass in the 100 to 300 bp size range, a finding which indicates that *Eco*RI generates the small difference in fragment sizes between these three combinations. The *Eco*RI/*Pst*I restriction endonuclease combination was also consistent across assemblies with similar peaks at approximately 750, 810 and 975 bp. However, this enzyme combination failed to yield a significant number of fragments across the entire fragment size window assessed compared to the profiles generated for the other three enzyme combinations. Taken together, this strongly suggested that the bulk of the in-silico digest was the result of the nuclease activity of *Alu*I, with minor contribution from *Eco*RI, and negligible fragment generation by *Pst*I in the assessed fragment size range of 100 to 1000 bp. Although the peak profile trends across assemblies were consistent, the fragment mass showed a degree of variability. This is likely attributable to variation in assembly size or completeness of each assembly. Similarly, assessment of the 1 to 10 kb fragment profiles suggested that the comparison between enzyme combinations was consistent across each assembly (Figure S1). More specifically, *Alu*I/*Pst*I produced slightly more fragment mass than did the *Alu*I/*Eco*RI/*Pst*I and *Alu*I/*Eco*RI combinations in the fragment size range of 1000 to 1500 bp. Beyond this fragment size range, the *Eco*RI/*Pst*I digestion profile indicated a greater amount of ‘actual’ fragments resulting from enzyme activity (Figure S1) due to their ‘rare cutting’ activity. 

With the in-silico analysis indicating that the restriction endonuclease combinations *Alu*I/*Eco*RI and *Alu*I/*Eco*RI/*Pst*I were ideal enzyme combinations for fragment generation in the desired range of 100 to 300 bp, evaluating the implementation of either single or double digestion reactions to determine endonuclease efficiency and the extent of gDNA digestion was next evaluated. In the case of each enzyme combination, the double digestion produced a greater inconsistency in fragmentation profile than did the single digest, as indicated by larger separation of profile lines (Figure S2). With the introduction of inconsistencies across each tested double digest combination with no discernible ‘positive’ change in profile peaks (i.e., development of new peaks within the sequencing range of 100 to 300 bp), it was concluded that a single digest with these enzyme combinations was sufficient to fully complete the digestion of *Cannabis* gDNA. Moreover, smear analysis of the 100 to 300 bp fragment size range (Figure 2C), indicated a greater concentration of fragments within this size range from the single digest, with the observed reduction in the concentration of 100 to 300 bp fragments in the double digests, potentially attributable to increased sample handling.

After determining that a single reaction was optimal for gDNA digestion, each restriction endonuclease combination was then compared for its ability to generate gDNA fragments ranging in length from 100 to 300 bp: the target range for fragment length for most short read sequencing platforms. The *Eco*RI/*Pst*I combination resulted in a large portion of fragments within the 700 bp to 40 kb range (Figure S2). Given the ‘rare cutting’ 6 bp recognition sequence of both the *Pst*I and *Eco*RI endonuclease for *Cannabis* gDNA, the generation of a pool of larger sized digestion fragments was expected and predicted by the in-silico modeling (Figure S1). The three remaining restriction endonuclease combinations returned similar peak profiles despite the inclusion of either *Eco*RI, *Pst*I, or both. This finding indicated that *Alu*I was largely responsible for the bulk of the observed endonuclease activity in each combination. Confirmation via smear analysis of the 100 to 300 bp fragment length range supports this observation, with the *Alu*I/*Eco*RI combination producing the greatest concentration of fragments within this range at 1777 nmol/L (Figure 2C), as well as the most consistent fragmentation concentration within this fragment length range, and between replicates (1777 and 1774 nmol/L, respectively). 

### 2.2. PCA and DAPC of Complete Variant Sets Can Distinguish Cannabis Varieties

To evaluate the genetic diversity, single nucleotide polymorphisms (SNPs) across all 83 plants assessed were identified, and following QC filtering, 3608 SNPs were retained. In a preliminary assessment of genetic diversity, Nei’s pairwise Genetic Distance was employed on the retained set of 3608 SNPs. This approach identified CBD1 and CBD2 as being the most distant from the other eight *Cannabis* varieties assessed (Table 1). Furthermore, CBD1 and CBD2 returned the lowest D value (0.0095) to indicate that these two varieties had fewer per locus allele differences than any other variety combination, and by extension, the lowest genetic differentiation. Genetic distance between Motavation and Blue Venom also exhibited a relatively low D value (0.0125) across the assessed varieties to similarly identify a close genetic relationship between these two *Cannabis* varieties. The average D value for Swiss Dream Auto plants (0.0573) was comparable to the D values exhibited by CBD1 (0.0575) and CBD2 (0.0569) plants when compared to the remaining varieties. It is important to note here that the Swiss Dream Auto variety was the only seed derived variety analyzed in this study, and also included the greatest number (*n* = 17) of individual plants assessed for the ten *Cannabis* varieties included in this study. This likely drove the observed increase in the relative value, given the inherent increase in genetic diversity of a seed grown plant, compared to clonal plants derived from cuttings. Overall, the average genetic distance values obtained for the remaining varieties were consistently low when compared to other studies in wheat, which describe D values from 0.1 to 0.5 across roughly 150 wheat accessions [14,15]. Nevertheless, this analysis highlighted the limited genetic distance between the *Cannabis* varieties CBD1 and CBD2 in particular, and to a lesser degree, the Motavation and Blue Venom varieties. 

**Figure 3:**
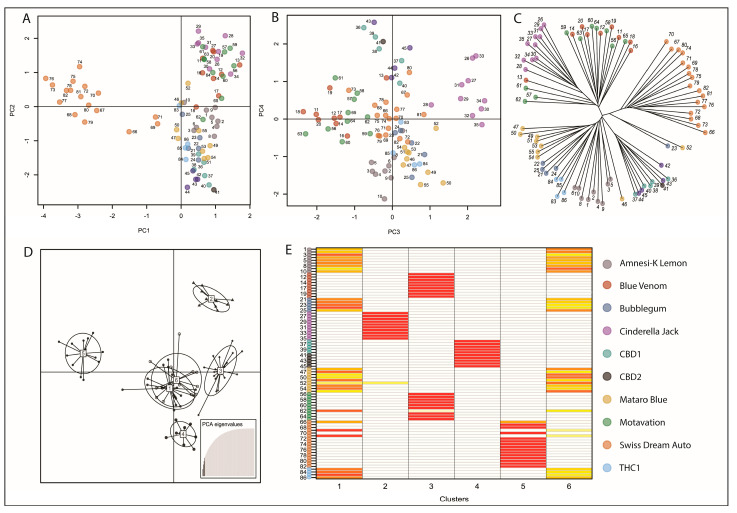
PCA and DAPC of variants found in genes identified to encode catalytic enzymes of the cannabinoid biosynthesis pathway. (**A**) Plot of the first 2 PC vectors; PC1 and PC2 (**B**) Plot of PC vectors, PC3 and PC4 (**C**) Unrooted phylogenetic tree demonstrated 6 discreet clusters based on PCA, which are also observed in the (**D**) DAPC plot. (**E**) Clusters 1 and 6 display extensive overlap, which is similarly observed in the assignplot where red shaded rectangles indicate high probability of correct assignment to each cluster, with the shading color spectrum passing through orange and yellow to white, representing a gradual reduction in probability. Color code indicates dot color and variety relationship throughout.

After identification of the SNP set, and the assessment of genetic distance, we next sought to intersect the SNP set with the 21 genes which encode the enzymes that catalyze each step of the cannabinoid synthesis pathway to determine the capability of reduced representation sequencing to initially isolate genes of interest, and to secondly, identify informative SNPs. A first assessment utilizing only coordinates that covered the region from the start codon to the stop codon of each annotated Purple Kush gene failed to align with any of the 3608 SNPs identified. Therefore, given that consequential polymorphisms are not restricted to coding sequences, we next extended the boundaries of the assessed genes by 500 kb up and downstream of the start and stop codon of each annotated gene to attempt to identify SNPs that may be co-inherited with genes of interest in a linkage block, or located in the promoter or other regulatory regions of each gene. This approach identified 102 of the 3608 SNPs which possessed some diagnostic utility in resolving different varieties. Retention of 4 axes in the PCA separated Swiss Dream Auto, Cinderella Jack, Blue Venom, Motavation, CBD1 and CBD2 from the remaining varieties, which was similarly reflected in a phylogenetic analysis (Figure 3C).

DAPC clustering also resolved the assessed varieties, with the exception of two individuals from Swiss Dream Auto and one individual from Motavation, as well as to group the remaining individuals into two mixed, indeterminate clusters (Figure 3D). Taken together, SNPs contained within the extended gene regions were not able to resolve assignment for the remaining individuals. A combination of filtering stringency, the restricted genomic region interrogated, and the nature of reduced representation sequencing, are causes for the lack of SNP retention in this region. This outcome also indicates that the *Alu*I and *Eco*RI restriction endonucleases used in combination for reduced representation, do not allow for deep enrichment of these regions. However, this finding does not discount the potential effectiveness of other enzyme combinations at producing fragments of the desired length and which are enriched for the coding sequences of the genes of the cannabinoid biosynthesis pathway.

**Figure 4:**
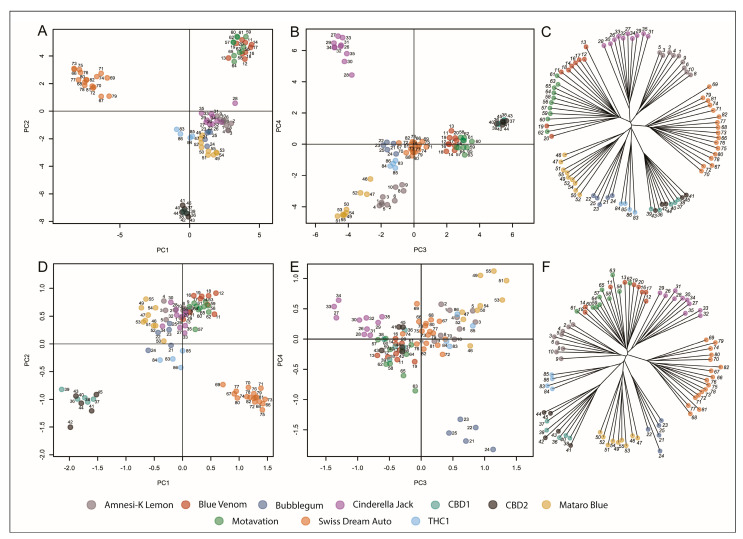
Principal component and phylogenetic analysis of 83 individual *Cannabis* plants. (**A**) PC1 and PC2 of the entire 3608 SNP dataset generated 4 distinct clusters. The central cluster of multiple varieties is resolved on PC3 and PC4 (**B**), with 8 clusters resolved on a phylogenetic tree (**C**). PCA of a reduced set of 172 SNPs shows variety grouping across (**D**) PC1/PC2 and (**E**) PC3/PC4 axes, as well as to produce the same 8 variety clusters as identified using the full 3608 SNP set in a phylogenetic analysis (**F**).

The inability to completely resolve the structure of the assessed varieties based on variants within putative gene and extended regulatory regions led us to next utilize the entire SNP dataset. Comparable in structure to the candidate gene analysis but significantly more defined, PCA using the full SNP dataset resolved the Swiss Dream Auto, CBD1, CBD2, Motavation, and Blue Venom varieties on the first 2 axes (Figure 4A,B). The remaining varieties were resolved on the final two axes of PCA (Figure 4D,E), and which were further confirmed by a phylogenetic analysis, where 8 defined clusters are evident on an unrooted tree (Figure 4C). Here the varieties CBD1 and CBD2 did not separate from one another, despite being described as distinct varieties. Blue Venom and Motavation also clustered together in these PCA and phylogeny analyses.

**Figure 5:**
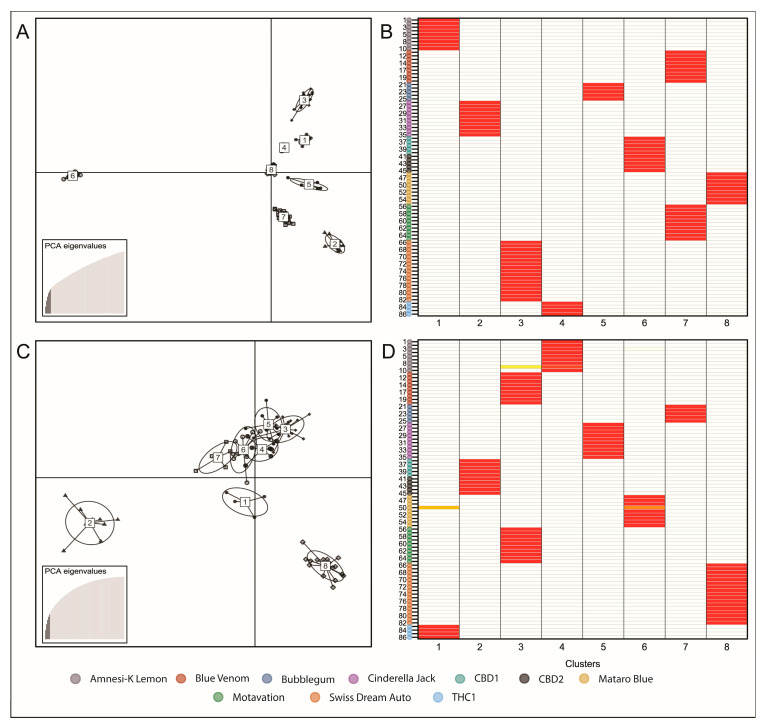
DAPC of two variant sets. (**A**) DAPC using the full set of 3608 SNPs produced 8 tight, and distinct clusters with each individual plant assigned to their respective cluster with high probability in an assignplot (**B**). (**C**) DAPC on a reduced set of diagnostic variants similarly resolved the assessed population into 8 distinct clusters, with all but 3 individual plants assigned to their cluster with high certainty in an assignplot (**D**).

DAPC resolved 8 tightly clustered groups, which were all assigned to their cluster or variety with certainty (Figure 5). The DAPC method is an iterative process that benefits from interactive user input and the balance lay in the potential of the method to overfit the model or lose informative data, which is influenced by the number of retained principal components (PCs) for DAPC. In each case, sufficient PCs were retained to account for approximately 40–50% of cumulative variance. This was enough to unambiguously infer group membership based on the total set of 3608 SNPs. Interestingly, CBD1 and CBD2 could not be partitioned into separate varieties under any iterative stringency of the available data and did not exhibit any level of group assignment uncertainty. These two varieties are phenotypically indistinguishable from one another as well as having been determined to possess similar secondary metabolite profiles. 

Similarly, while the *Cannabis* varieties Motavation and Blue Venom do display distinct phenotypes, they were not identified as separate varieties using the SNP-filtering approach utilized here. Notably, Motavation and Blue Venom both originated from a common seed supplier and produce similar cannabinoid profiles (G.M.S, personal communication), and potentially highlights the limitations inherent in classification strategies for *Cannabis* varieties based on phenotypic traits and should be supplemented with robust genomic annotation. 

### 2.3. Cannabis Varieties Can Be Identified by a Minimal Number of SNPs Highly Associated with the LTR Class of Retroelement

Following the discreet partitioning of individuals into varieties based on the full number of SNPs of the entire dataset, the number of SNPs was reduced to a minimal set of diagnostically informative SNPs capable of assigning individual plants to their respective varieties. We further filtered the full set of SNPs to exclude SNPs which were positioned within 1 Mb of another identified SNP to identify and retain only the SNPs which were likely to be inherited independently. This filtering approach produced a final set of 172 SNPs. PCA showed similar clustering patterns to those observed in the previous analyses using the full set of SNPs, and furthermore, phylogenetic analysis also produced a clustering model which was highly reflective of a clustering model generated using the full dataset (Figure 4). Additionally, DAPC clustering identified 8 distinct groupings, and aside from two individual plants, assigned membership of each assessed plant to their respective varietal group with certainty (Figure 5). Although these two individual plants could be assigned to their respective varietal clusters, they also presented a level of uncertainty towards other clusters. Nevertheless, the analyses using the reduced set of SNPs displayed a diagnostic power analogous to that of the analyses performed using the full set of 3608 SNPs and can thus be used to partition individuals into variety classification accurately.

Although the candidate gene associated SNPs were not able to fully resolve the population structure of the assessed *Cannabis* varieties, an alternate and reduced set comprised of 172 SNPs was successfully employed to do so. Further interrogation of the 172 SNPs to determine their genomic location revealed that these variants had a high association with the long terminal repeat (LTR) retroelement class of transposon; only 8.1% (*n* = 14) of SNPs of the reduced dataset were mapped to either within a protein-coding gene sequence (*n* = 7), or within the 6 kb of the chromosomal DNA sequence which immediately flanks a protein-coding gene (*n* = 7). In contrast, 91.9% of the reduced set of SNPs (*n* = 158) mapped to within a reported LTR retroelement (*n* = 121), or to a 3 kb region immediately flanking a reported LTR retroelement (*n* = 37). While the relationship between LTR sequences and cannabinoid synthesis is unclear, intergenic and ‘junk’ DNA is frequently associated with regulatory enhancer or repressor elements, or to the maintenance of chromatin structure, both of which are known to effect transcriptional dynamics.

## 3. Conclusions

Despite the historically pervasive use of *Cannabis* and the pharmacologically significant applications of its metabolites, scientific research and classification rigor of this species remains in its infancy. Indeed, within the *Cannabis* industry, varieties may be arbitrarily designated as ‘novel’ with little scientific information available regarding the ancestral genetics of each plant and the extreme level of outcrossing or inbreeding that has occurred, often in recent decades. For example, a recent metabolomic analysis of 89,923 samples of dried *Cannabis* flowers from the Canadian market revealed a disparity between product labeling and secondary metabolite profiles, and directly stemming from this finding, called for a classification and naming system to more accurately represent the diversity of *Cannabis* varieties [16]. One recent attempt at this was an analysis of terpene synthase genes which suggested using a set of genetic markers associated with the terpenes as preferable to the modern *Cannabis* culture labeling of varieties as either ‘*sativa*’ or ‘*indica*’, based on aroma [17]. 

To address the need for better genetic classification methods, here we successfully applied a genome reduction sequencing approach to identify SNPs associated with cannabinoid synthesis and strain classification across multiple *Cannabis* varieties. Notably, a minimal number of SNPs (*n* = 172) was able to achieve a high degree of diagnostic accuracy, effectively grouping varieties according to their cannabinoid profiles. Moreover, the small number of regions also offers the possibility to design a small and highly targeted resequencing assay which could be deployed cost-effectively to rapidly screen *Cannabis* plants on the market at scale. 

While the plasticity of *Cannabis* phenotype and chemotype as a response to environmental conditions must also be considered as a variable for variety classification, recent studies suggest it is one that can be nullified by DNA level analysis. For example, a recent assay based on the use of SNPs associated with secondary metabolite synthesis and fiber production was successfully applied to distinguish ’fiber’ from ‘drug’ type *Cannabis* varieties [18]. A second study that used a whole genome analysis approach reported the successful classification of *Cannabis* varieties into THC/CBD content groupings using multiallelic SNPs [19]. Both reports also call for, and can be utilized as, genomics-based classification within the industry. 

One notable finding of this study was the prominent association of the reduced set of SNPs with the LTR class of retroelement, with nearly 92% of the most diagnostically informative SNPs being associated with these elements. The ability for this specific set of SNPs to provide information on population structure and variety classification was somewhat unexpected, however transposable-element (TE)-based assays have been successfully utilized previously for phylogenetic studies in other plant species [20,21]. How LTRs specifically influence cannabinoid synthesis and regulation is unclear from the current analysis, but there are some possibilities. While retrotransposons are usually rendered ‘transcriptionally silent’ via DNA methylation [22], and have the potential to be highly deleterious if insertion occurs in the regulatory or coding regions of a protein-coding gene, subsets of TEs will retain their activity and can be important factors in the ongoing evolution of complex genomes [23]. As part of this process, TEs have also been shown to add to the complexity of the regulation of the expression of nearby genes following their proximal movement [24,25], and similar processes may explain their association with cannabinoid production in the *Cannabis* varieties examined here. For example, the polymorphisms which result from TE activity were analyzed in clonally propagated *Vitis vinifera L.* (common grapevine), with TE chromosome relocation suggested to be the result of continued vegetative propagation [26]. Similarly, TE movement has been associated with the development of commercially significant traits in agricultural species [27,28], in which heavy selection pressure may have played a role in TE activation. Currently, no study has examined the LTR class of retroelement in detail in *Cannabis*, but given the significance of clonal propagation and the application of selection pressure as part of the process of cultivating *Cannabis*, this TE class may be more active in the *Cannabis* genome than in the nuclear genome of other plant species. As a result, TEs may have potentially played a more prominent role in driving phenotypic variation—and by extension CBD and THC pathway modification—in *Cannabis* cultivation. If this is determined to be the case in *Cannabis*, then polymorphism of this class of TE will form a viable target for variant analyses. Nonetheless, the data presented in this study indicates that further investigation of the possible role that TE movement has played in floral morphogenesis and secondary metabolite biosynthesis in *Cannabis* is warranted.

In summary, to further legitimize the *Cannabis* industry in terms of commercial cultivation and medicinal therapy, strict characterization of varieties should be established in the form of genomic identification that supports standardized metabolomics. Here we show a minimal cost, reduced representation sequencing method capable of discriminating *Cannabis* varieties, utilizing a limited SNP subset highly associated with the LTR class of retrotransposon. This approach, or similar methods, could be used by *Cannabis* growers to determine if their varieties are distinct from other varieties available on the market. Moving forward into an era of *Cannabis* based medicinal treatments, standardized classification of varieties must be ubiquitous and embedded throughout the industry from the seed onwards. Scientific quantification of plant traits, genomic, metabolomic, or otherwise, should be available to reliably inform growers, researchers, and other end users of *Cannabis* derived products of the true nature of the product to a standard demanded of any pharmaceutical product. 

## 4. Materials and Methods

### 4.1. In-Silico Restriction Digest of Cannabis Genome Assemblies

Four publicly available genome assemblies were used in this study, and included: (1) THCA producing, Purple Kush, (2) hemp variety, Finola [29], (3) mixed THCA/CBDA producing variety, Jamaican Lion DASH [30], and (4) high CBDA producing, CBDrx [31], with all four assemblies having employed some form of long read sequencing for generation of their data. Using Python (version 3.8.7), we developed an in-house script to assess the assemblies based on the restriction enzyme (RE) sequences and report the resulting fragment sizes, cut sites, and sequences. The fragment size limitations were set to more accurately replicate sizes suited to Illumina short read sequencing platforms. Using Seaborn [32] and Matplotlib [33] libraries in Python, the in-silico digests were graphed to qualitatively assess profile similarities across assemblies and digest conditions. Using the Tuxedo suite of tools, index genomes for each of the assemblies were created using Bowtie2 (version 2.3.0) [34], and via the use of an additional Python script, the restriction fragments were mapped to the indexed assemblies and reported on the mapping metrics. Following this, BEDtools (version 2.17.0) [35] was used to assess intersection of the restriction fragments with known or putative genes involved in cannabinoid biosynthesis from available annotations.

### 4.2. Plant Material and DNA Extraction

Ten *Cannabis* varieties comprising 87 plants were used in this study and included 6 high THC producing varieties which were represented by 55 individual plants. More specifically, the 55 high THC producing varieties assessed included, 10 Amnesi-K Lemon plants, 10 Blue Venom plants, 10 Cinderella Jack plants, 10 Mataro Blue plants, 10 Motavation plants, and a further 5 plants from an additional variety described here as THC1 to protect the intellectual property of the variety. Additionally, gDNA was extracted from a further 4 high CBD producing varieties which were represented by 32 individual plants. Moreover, the high CBD population of plants included, 5 Bubblegum plants, 17 Swiss Dream Auto plants, 5 CBD1 plants, and 5 CBD2 plants, with the CBD1 and CBD2 varieties also named as such in this study to protect the intellectual property of each variety. For all plant varieties, gDNA was extracted from young leaves using a DNeasy Plant Pro Kit (Qiagen, Hilden, Germany) exactly as according to the manufacturer’s instructions. Sample concentrations were assessed using a NanoDrop One (Thermo Scientific, Waltham, MA, USA) spectrophotometer.

### 4.3. Restriction Endonuclease Digestion of Genomic DNA

Three REs were selected for assessment based upon their nuclease activity in the same buffer and temperature conditions. Four RE combinations (3 double enzyme, 1 triple enzyme) were tested on pooled *Cannabis* gDNA to ascertain the best digestion efficiency and fragment pool generation for downstream sequencing. The combinations included (1) *Alu*I and *Eco*RI; (2) *Alu*I and *Pst*I; (3) *Eco*RI and *Pst*I; and (4) *Alu*I, *Eco*RI, and *Pst*I, which were run in duplicate with half of the samples digested twice to confirm that the reaction had reached completion. 

### 4.4. Sample Preparation and DNA Sequencing

Four hundred nanograms (400 ng) of each gDNA sample was digested with 10 units of *Alu*I and *Eco*RI for 60 min at 37 °C with shaking at 600 rpm, in a total reaction volume of 25 µL. Post digestion, each sample was cleaned using AgencourtXP beads (Beckman Coulter, Brea, CA, USA) to separate large from small sized fragments. In brief, 42 µL of water was added to the 25 µL reaction, followed by the addition of 40 µL of XP beads (to achieve a 0.6 bead to sample ratio (*v*/*v*)). After thorough mixing and the placement of each sample on a magnetic rack, the supernatant was transferred to a new tube, while the larger sized DNA fragments (i.e., >1 kb) were retained by the beads in the original reaction tube. An additional 60 µL of XP beads was then added to the transferred supernatant (to give a final 1.8 bead to sample ratio (*v*/*v*)), followed by mixing, and washing twice with 70% ethanol (*v*/*v*), and subsequent elution in low TE.

Libraries of the smaller sized DNA fragments were prepared using UltraII End Repair and Ligation kits (New England Biolabs, Ipswich, MA, USA), with custom Y-adaptors. Post-adaptor ligation, samples were cleaned with XP beads using a 1.5 bead to sample ratio (*v*/*v*), and this was followed by 4 cycles of PCR using Q5 2X HotStart MasterMix (New England Biolabs, Ipswich, MA, USA). Library profiles were assessed using a Labchip GX (PerkinElmer, Waltham, MA, USA), followed by pooling equal masses. Sequencing was performed on an Nextseq 500 (Illumina, San Diego, CA, USA), using a Mid 300 Kit in 150 PE configuration.

### 4.5. Read Mapping

To assess preliminary mapping metrics, one set of paired-end fastq files from each of the ten plant varieties was selected and mapped against each of the four genome assemblies using HISAT2 (version 2.1.1.) [36] with --no-spliced-alignment and --no-softclip options. The mapping metrics were qualitatively assessed for each sample and assembly, with consideration given to the overall alignment, unmapped, uniquely mapping, and multi mapping reads, as well as the genome assembly size. All 87 samples were then mapped to the Purple Kush genome assembly using HISAT2 with the BAM file output of mapping reads then sorted using SAMtools (version 1.6) [37] sort. 

### 4.6. Determination of In-Silico and Sequenced Fragment Overlap

Using the in-house custom in-silico restriction digest Python script, a bed file was generated consisting of 100 to 600 bp fragment coordinates resulting from an EcoRI/AluI enzyme digestion combination on the Purple Kush genome to ostensibly reflect those fragments predicted to be sequenced in actuality. A complement bed file consisting of the remaining genomic regions not resulting from the EcoRI/AluI digest was then generated using BEDtools complement. These bed files were used in conjunction with the previously generated bam files from each of the 87 samples in BEDtools multicov to assess the overlap between the in-silico and sequenced fragments, as well as the remaining genomic regions to determine on/off target enrichment. 

### 4.7. Variant Calling

Variant analysis of HISAT2 generated and sorted BAM files was conducted using the germline workflow of Strelka2 (version 2.9.10) [38] in combination with the Purple Kush genome assembly as the reference sequence. Using the SuperCann *Cannabis* Multiomics Database [39] resources, including the GenomeBrowser, BLAST, and assembly annotation files for the Purple Kush assembly, a BED file was generated of predicted start and stop site coordinates from the SuperCann gene annotation for 21 genes involved in cannabinoid biosynthesis, and the range extended up and downstream of these genes by 500 kb (Table S1). Using VCFtools (version 0.1.8a) [40], the multi-variant VCF output file from Strelka2 was filtered to exclude poorly sequenced individuals using an error count script from dDocent [41], which removed 4 samples from the population to produce a final count of 83. Next the file was filtered to include sites with a minimum read depth of ≥5, a minor allele count ≥ 3, minimum quality score ≥ 30, maximum missing data per site of 0.80, minor allele frequency 0.05, and a minimum mean depth across all individuals ≥ 25. For the variant file of cannabinoid pathway gene loci with extended range, filtering parameters were a minimum read depth of ≥5, minor allele count ≥ 3, minimum quality score ≥ 30, and the minor allele frequency at 0.05. A VCF of cannabinoid gene coordinates was then generated from this using BEDtools intersect. Additionally, a third VCF was generated containing a reduced subset of SNPs by filtering the final, full variants VCF to a minor allele frequency of 0.35.

### 4.8. Principal Component Analysis and Discriminant Analysis of Principal Components

To identify group clustering within the sequenced population, Principal Component Analysis (PCA) and Discriminant Analysis of Principal Components (DAPC) [42] was used in R through the packages ‘adegenet’ [43,44] and ‘vcfR’ [45]. To store binary SNPs for computational efficiency, VCFs were converted to genlight objects using the vcfR2genlight function in vcfR to store large amount of binary SNP data to complete the PCA. Within the adegenet package, the find.clusters function, using *k*-means clustering, was used to identify clusters from the PCA where the number of clusters is chosen based on the lowest Bayesian information criterion (BIC) value or where an ‘elbow’ occurs in the plot. Enough principal components (PCs) were retained to account for approximately 80% of the variance in the PCAs performed. Then using the dapc function, enough PCs were interactively chosen to account for approximately 40% of the data in all three cases, after which all linear discriminants were retained. 

### 4.9. Calculating Pairwise Genetic Distance between Varieties Using Nei’s Genetic Distance 

To calculate pairwise genetic distance between varieties, a population ID was assigned to each individual in the previously generated genlight object of 3608 SNPs using the gl.define.pop function in the R package, ‘dartR’ (version 1.9.4) [46]. This variety assignment was based on the initial designation of 10 distinct varieties. A genetic distance matrix was then produced on the genlight object using the stamppNeisD function in the R package, ‘StAMPP’ (version. 1.6.3) [47].

## Figures and Tables

**Table 1:** Analysis of Nei’s genetic difference across 10 *Cannabis* varieties. Green indicates a greater genetic difference to the compared variety, while red indicates a reduced difference.

	AK	BV	Bu	CJ	CBD1	CBD2	MB	Mot	SDA	THC1
AK	0	0.0388	0.0444	0.0463	0.0575	0.0575	0.0349	0.0407	0.0538	0.0423
BV	0.0388	0	0.052	0.0475	0.0697	0.0695	0.0514	0.0125	0.0556	0.0573
Bu	0.0444	0.052	0	0.0472	0.0589	0.0573	0.0425	0.0495	0.057	0.0506
CJ	0.0463	0.0475	0.0472	0	0.0664	0.0662	0.0478	0.0503	0.0572	0.0529
CBD1	0.0575	0.0697	0.0589	0.0664	0	0.0095	0.0599	0.0689	0.0665	0.06
CBD2	0.0575	0.0695	0.0573	0.0662	0.0095	0	0.0597	0.0673	0.066	0.0594
MB	0.0349	0.0514	0.0425	0.0478	0.0599	0.0597	0	0.0536	0.0552	0.0455
Mot	0.0407	0.0125	0.0495	0.0503	0.0689	0.0673	0.0536	0	0.0531	0.0538
SDA	0.0538	0.0556	0.057	0.0572	0.0665	0.066	0.0552	0.0531	0	0.0512
THC1	0.0423	0.0573	0.0506	0.0529	0.06	0.0594	0.0455	0.0538	0.0512	0

## Data Availability

The sequence data generated in this study have been deposited in the NCBI database under BioProject PRJNA894796. In-house Python scripts for genome fragmentation modelling has been submitted to a GitHub repository and can be found at https://github.com/uonoul/reduced-representation-model.

## Supplementary Materials

The following supporting information can be downloaded at: https://www.mdpi.com/article/10.3390/ijms232314531/s1, Figure S1: In-silico digest profiles of 4 Cannabis assemblies with 4 restriction enzyme combinations from 1 to 10 kb fragment size.; Figure S2: Single and double digest outcomes for restriction enzyme combinations; Table S1: Cannabinoid biosynthesis gene coordinates including additional 500 kb up and downstream.
