# Supplementary material for: Genetic Variants Associated with Long-Terminal Repeats Can Diagnostically Classify Cannabis Varieties
Source: Int J Mol Sci. 2022 Nov 22;23(23):14531. doi: 10.3390/ijms232314531 (PMC9735643; doi:10.3390/ijms232314531)
Supplement: Supplementary file 1 [file ijms-23-14531-s001.zip › ijms-2037205-supplementary.pdf]

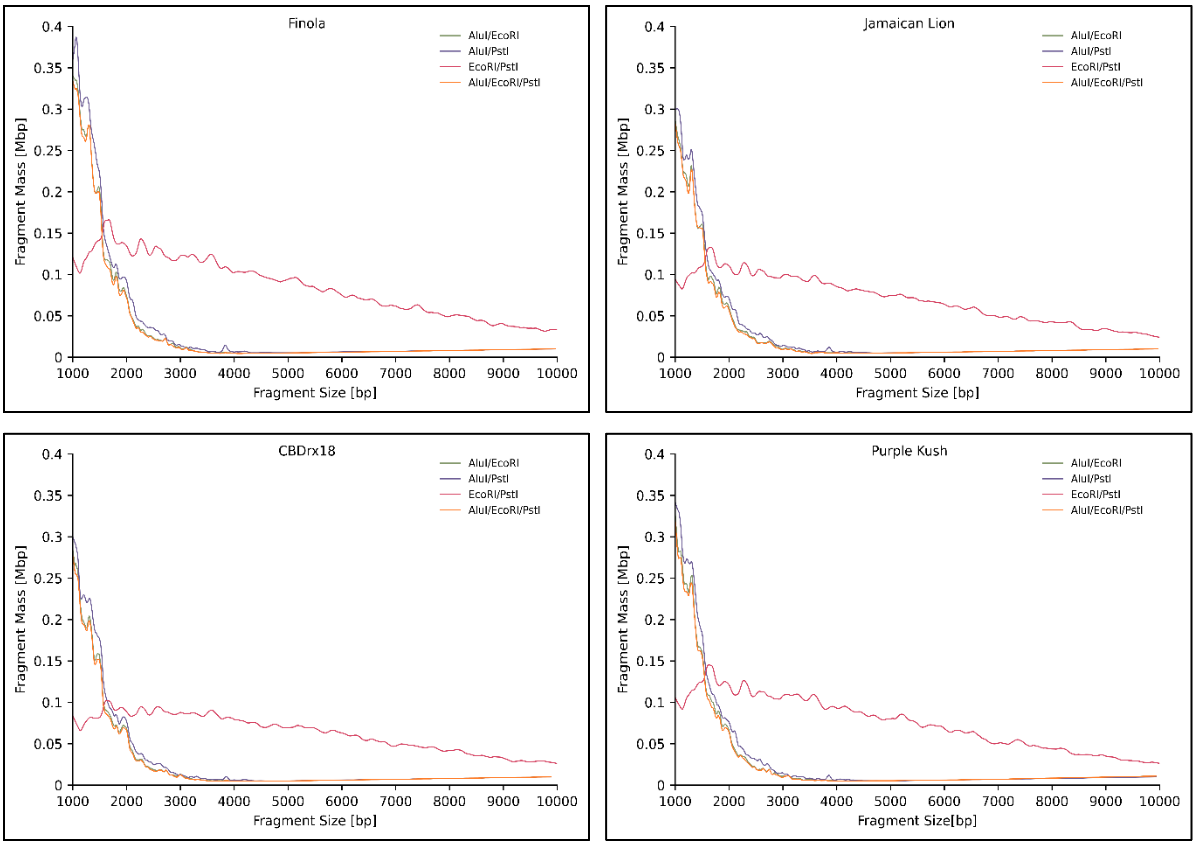

Figure S1. In-silico digest profiles of 4 Cannabis assemblies with 4 restriction enzyme combinations from 1 to 10kb fragment size. Four Cannabis assemblies, Finola (FN), Purple Kush (PK), CBDrx18 (CBD), and Jamaican Lion DASH (JLD), were digested in silico with four restriction endonuclease combinations, including the AluI/EcoRI, AluI/PstI, EcoRI/PstI, and AluI/EcoRI/PstI combinations, to examine the fragmentation profiles between 1kb to 10kb.

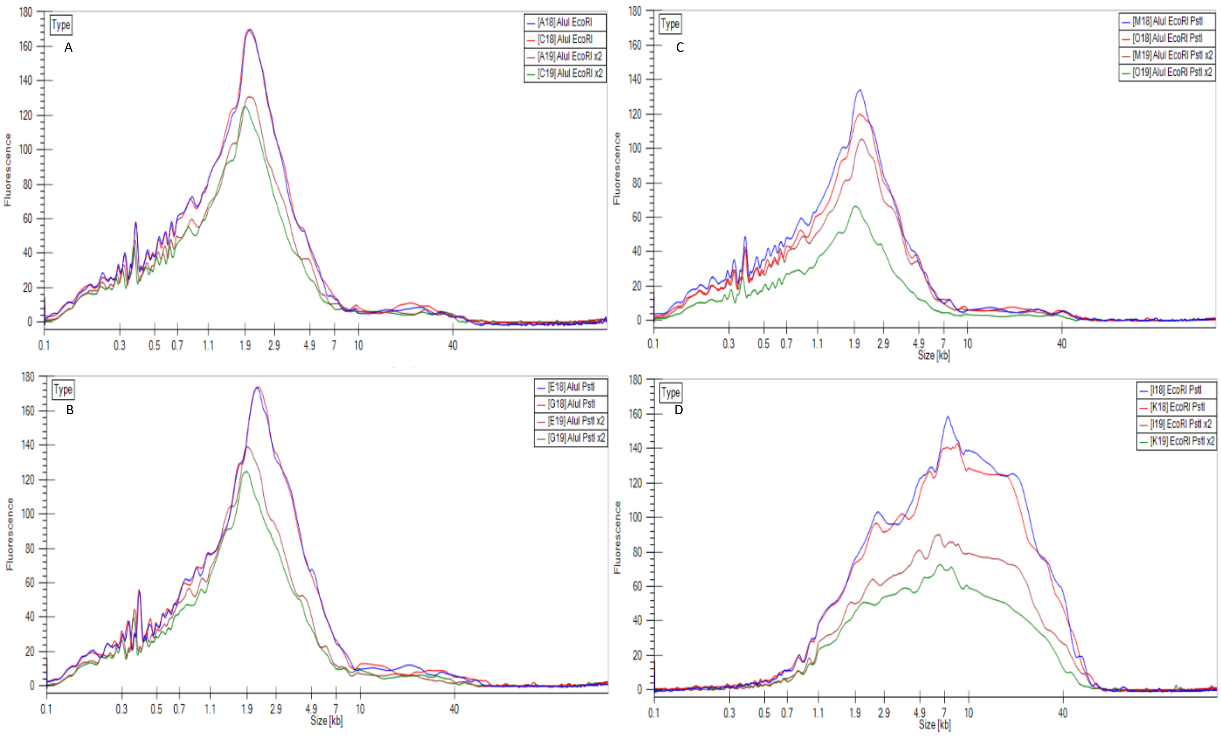

Figure S2. Single and double digest outcomes for restriction enzyme combinations. *Cannabis* gDNA was digested with four restriction endonuclease combinations, including the AluI/EcoRI, AluI/PstI, EcoRI/PstI, and AluI/EcoRI/PstI combinations and the fragmentation profiles were assessed using a LabChip GX.

Table S1. Cannabinoid biosynthesis gene coordinates including additional 500kb up and downstream.

Chromosome	Start - 500kb	Start	Stop	Stop + 500kb	Gene
AGQN03000389.1	0	175327	182874	682874	HDR
Chr1	55925011	56425011	56430287	56930287	PKS
Chr1	40300096	40800096	40808418	41308418	HMGS
Chr1	51134888	51634888	51644792	52144792	PMK
Chr2	55180934	55680934	55686341	56186341	GPPS ssu
Chr2	68434029	68934029	68955726	69455726	DXS1
Chr3	25894662	26394662	26401664	26901664	MK
Chr4	8524298	9024298	9034714	9534714	DXR
Chr4	37803447	38303447	38318838	38818838	AAE1
Chr5	622073	1122073	1126936	1626936	GPPS ssu2
Chr5	59163665	59663665	59674108	60174108	HDS
Chr5	3247761	3747761	3757729	4257729	OAC
Chr6	18526191	19026191	19034144	19534144	MPDC
Chr6	5048446	5548446	5556699	6056699	FPPS
Chr6	61582305	62082305	62093091	62593091	CBDAS
Chr7	9571611	10071611	10080024	10580024	CMK
Chr7	7563636	8063636	8075446	8575446	ACCa
Chr9	0	6779663	6785415	630053	GPPS lsu
Chr9	6279663	123153	130053	7285415	IPP
Chr9	7816822	8316822	8323797	8823797	HMGR
Chr9	28013194	28513194	28520778	29020778	MCT
